# To protect or reject

**DOI:** 10.7554/eLife.03374

**Published:** 2014-06-17

**Authors:** Mary Munson

**Affiliations:** 1 **Mary Munson** is in the Department of Biochemistry and Molecular Pharmacology, University of Massachusetts Medical School, Worcester, United Statesmary.munson@umassmed.edu

**Keywords:** membrane, SNARE, Docking, HOPS, lysosome, Golgi, *S. cerevisiae*

## Abstract

A protein known for its role in dismantling faulty SNARE complexes can also help to maintain complexes that have formed properly during membrane fusion.

**Related research article** Lobingier BT, Nickerson DP, Lo S-Y, Merz AJ. 2014. SM proteins Sly1 and Vps33 co-assemble with Sec17 and SNARE complexes to oppose SNARE disassembly by Sec18. *eLife* **3**:e02272. doi: 10.7554/eLife.02272

**Image** Proteins that ensure proper membrane fusion

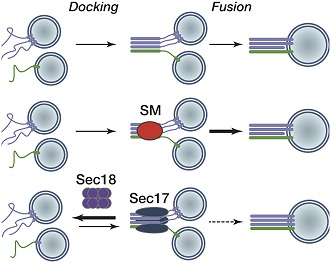

One of the hallmarks of eukaryotic cells is that they contain a number of distinct compartments called organelles. Contained within a membrane that is similar to the plasma membrane that encloses the cell itself, each organelle performs specific functions within the cell. Many macromolecules, such as proteins and lipids, must be moved between these organelles, or from an organelle to the cell surface to be released from the cell. These macromolecules are transported by membrane-bound structures called vesicles. Once at its target, a vesicle releases its contents by fusing its membrane to the membrane of the target. This fusion requires SNARE proteins to be present on both the vesicle and the target membranes. The individual SNARE proteins zipper together, forming remarkably stable *trans*-SNARE complexes that provide the energy needed to fuse the membranes (Jahn and Scheller, 2006; Rizo and Sudhof, 2012).

Membrane fusion is highly regulated by several conserved families of proteins. These regulatory proteins ensure that the SNARE membrane fusion machinery only engages and fuses the correct membranes at the right place and the right time. Identifying and understanding this molecular machinery has fascinated cell biologists, biochemists and structural biologists for many decades, and three leaders in the field–James Rothman, Randy Schekman and Thomas Südhof—shared the 2013 Nobel Prize in Physiology or Medicine. Now, in *eLife*, Alexey Merz and colleagues at the University of Washington School of Medicine—including Braden Lobingier as first author—shed new light on this process (Lobingier et al., 2014).

Many opportunities exist during membrane trafficking for both appropriately and inappropriately mixed SNARE complexes to form (see, for example, Furukawa and Mima, 2014), and cells go to great lengths to prevent incorrect fusion events. Cells regulate where and when SNARE complexes form in many ways: these include restricting the location of active SNARE proteins to fusion sites, stimulating fusion when the correct SNARE complexes are present, and disassembling incorrect SNARE complexes before fusion can occur.

After fusion and cargo delivery, the ATPase Sec18 (which is the yeast version of NSF in humans) and its partner Sec17 disassemble SNARE complexes so they can be recycled and re-used in subsequent fusion events (Chang et al., 2012). However, Sec18 and Sec17 are able to recognise and disassemble SNARE complexes at any step during trafficking, whether before or after fusion. This presents a dilemma for the cell—how can the premature disassembly of the correct SNARE complexes be prevented?

The Sec1/Munc18 (SM) family of proteins was thought to be able to ‘proofread’ the SNARE complexes, checking that the correct complexes have formed (Starai et al., 2008). However, this had not been directly demonstrated with SM proteins alone. The function of SM proteins in the cell remains confusing and controversial, as they can interact with individual SNARE proteins, as well as assembled SNARE complexes (Carr and Rizo, 2010). Four families of SM proteins regulate various steps in the vesicle trafficking pathways: Sly1, Sec1/Munc18, Vps45 and Vps33. By binding to SNAREs in several different ways, SM proteins play a number of roles in regulating where and when the complexes form. These roles include functioning as SNARE chaperones, SNARE inhibitors and SNARE activators, and also as stimulators of SNARE-mediated artificial vesicle fusion reactions.

Through a combination of biochemical reconstitution experiments using purified proteins, and genetic experiments in yeast, Lobingier et al. reveal a surprising and satisfying finding. Two SM proteins—Sly1 and Vps33—each collaborate with the disassembly factor Sec17 to proofread the SNARE complexes, protecting the correct SNARE complexes from being disassembled by Sec17 and Sec18 (Figure 1A). Moreover, both Sly1 and Vps33 bind poorly to assembled SNARE complexes, but Sec17 helps to load these SM proteins onto the correct SNARE complexes. In the case of inappropriately paired SNAREs, or SNARE complexes that form in the wrong location, it is likely that Sec17 will not load the SM protein onto these complexes and so they will quickly be disassembled (Figure 1B). Thus, Sec17 takes on a new role: protecting SNARE complexes from itself.

**Figure 1.**
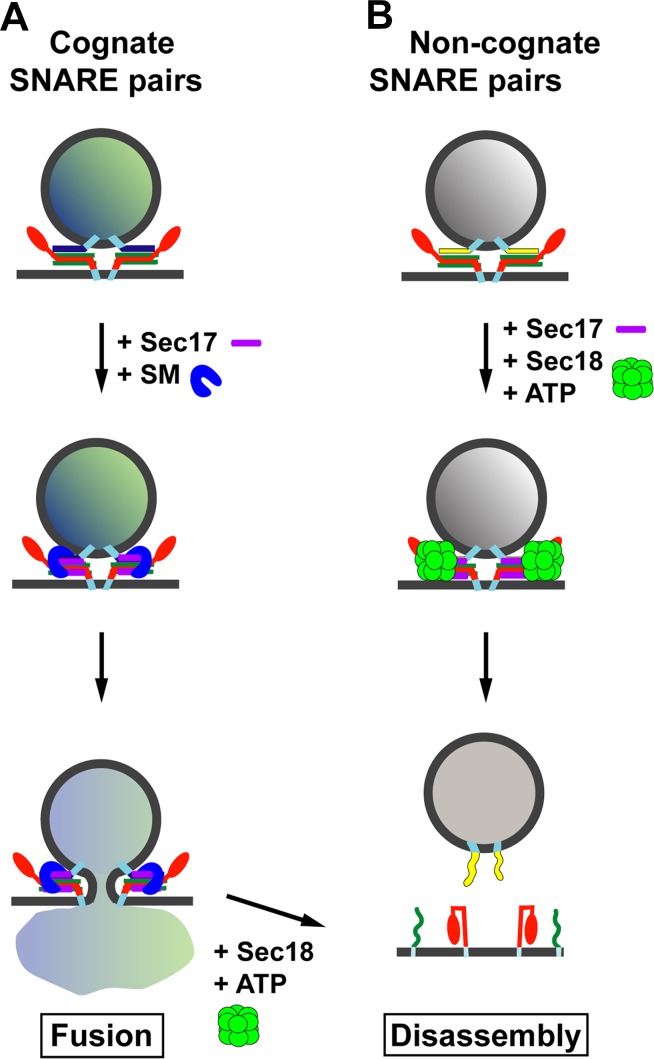
Model for the role of Sec17 in protecting SNARE complexes from premature disassembly, and for facilitating the disassembly of incorrectly formed SNARE complexes. A vesicle releases its contents by fusing with the membrane of its target. For the vesicle to dock with its target, the SNARE proteins on the target membrane (red and green) must form *trans*-SNARE complexes with the SNARE proteins on the vesicle membrane (dark blue in (**A**); yellow in (**B**)). The SNARE adaptor protein Sec17 (purple) recognizes the SNARE complexes, and up to three copies of Sec17 can bind to the complex. If a correct SNARE complex has formed (**A**), Sec17 then recruits an SM protein (blue arch): this helps to promote membrane fusion, and the vesicle can release its cargo into the target. However, if the complex is incorrect (**B**), Sec17 engages the ATPase Sec18 (green) to disassemble the SNARE complex before fusion can occur: when this happens the vesicle cannot release its cargo into the target. In (**A**), after membrane fusion occurs, Sec17 and Sec18 take apart the SNARE complexes for use in subsequent fusion events.

Raising the temperature of the reaction increases the rate at which the SM proteins bind to the SNARE complexes. This finding suggests that the SM proteins may undergo a transition from a conformation suitable for binding to a single SNARE protein called syntaxin, to one that interacts with SNARE complexes—an idea previously suggested for other SM proteins (for example, see Christie et al., 2012).

Mechanistically, Sec17 acts as a general SNARE adaptor protein, binding SNARE complexes and deciding whether to recruit an SM protein for fusion, or Sec18 for disassembly. After fusion occurs, Sec17 presumably favours the recruitment of Sec18 for prompt disassembly and recycling of the SNARE complexes. How Sec17 makes these critical decisions in cooperation with the SM proteins will no doubt be revealed by further biochemical and structural investigations. To this end, recent results suggest that, in the absence of SM proteins or Sec18, a protein called α-SNAP (which is the mammalian version of Sec17) interferes with SNARE zippering and membrane fusion when it binds with a SNARE complex (Park et al., 2014). Sec17 acts as if it is poised and awaiting the binding of an SM protein or Sec18 for its subsequent activity.

Is the Sec17-SM proofreading activity a universal feature of Sec17 and all the SM proteins? It seems likely that SNARE proofreading is a critical mechanism used at all steps to ensure the correct membranes fuse together. The fact that several of the SM proteins can bind SNARE complexes tightly in the absence of Sec17 suggests, however, that Sec17 might be dispensable for proofreading at some stages in vesicle trafficking. Furthermore, the effects on this process of SNARE transmembrane domains and the presence of lipids, plus the role of the various specific tethering complexes that bind to both SNAREs and SM proteins (Hong and Lev, 2014) still await discovery.
